# *Paris Polyphylla* Inhibits Colorectal Cancer Cells via Inducing Autophagy and Enhancing the Efficacy of Chemotherapeutic Drug Doxorubicin

**DOI:** 10.3390/molecules24112102

**Published:** 2019-06-03

**Authors:** Liang-Tzung Lin, Wu-Ching Uen, Chen-Yen Choong, Yeu-Ching Shi, Bao-Hong Lee, Cheng-Jeng Tai, Chen-Jei Tai

**Affiliations:** 1 Department of Microbiology and Immunology, School of Medicine, College of Medicine, Taipei Medical University, Taipei 11042, Taiwan; ltlin@tmu.edu.tw; 2 Graduate Institute of Medical Sciences, College of Medicine, Taipei Medical University, Taipei 11042, Taiwan; 3 School of Medicine, Fujen Catholic University, New Taipei City 24205, Taiwan; m002047@ms.skh.org.tw; 4 Department of Hematology and Oncology, Shin Kong Wu Ho-Su Memorial Hospital, Taipei 11042, Taiwan; 5 Division of Hematology and Oncology, Department of Internal Medicine, Taipei Medicine University Hospital, Taipei 11042, Taiwan; chenyen@tmu.edu.tw (C.-Y.C.); jasmineycs@yahoo.com.tw (Y.-C.S.); f96b47117@ntu.edu.tw (B.-H.L.); cjtai@tmu.edu.tw (C.-J.T.); 6 Division of Hematology and Oncology, Department of Internal Medicine, School of Medicine, College of Medicine, Taipei Medical University, Taipei 11042, Taiwan; 7 Department of Chinese Medicine, Taipei University Hospital, Taipei 11042, Taiwan; 8 Traditional Herbal Medicine Research Center, Taipei Medical University Hospital, Taipei 11042, Taiwan; 9 Department of Obstetrics and Gynecology, School of Medicine, College of Medicine, Taipei Medical University, Taipei 11042, Taiwan

**Keywords:** folk medicine, DLD-1 cells, doxorubicin, chemotherapy, drug resistance

## Abstract

Colorectal cancer is one of the most common cancers worldwide and chemotherapy is the main approach for the treatment of advanced and recurrent cases. Developing an effective complementary therapy could help to improve tumor suppression efficiency and control adverse effects from chemotherapy. *Paris polyphylla* is a folk medicine for treating various forms of cancer, but its effect on colorectal cancer is largely unexplored. The aim of the present study is to investigate the tumor suppression efficacy and the mechanism of action of the ethanolic extract from *P. polyphylla* (EEPP) in DLD-1 human colorectal carcinoma cells and to evaluate its combined effect with chemotherapeutic drug doxorubicin. The data indicated that EEPP induced DLD-1 cell death via the upregulation of the autophagy markers, without triggering p53- and caspase-3-dependent apoptosis. Moreover, EEPP treatment in combination with doxorubicin enhanced cytotoxicity in these tumor cells. Pennogenin 3-*O*-beta-chacotrioside and polyphyllin VI were isolated from EEPP and identified as the main candidate active components. Our results suggest that EEPP deserves further evaluation for development as complementary chemotherapy for colorectal cancer.

## 1. Introduction

*Paris polyphylla* is a well-known herbal medicine used in China and Taiwan, primarily to treat fevers, headaches, burns, and wounds, and for neutralizing snake poison [1]. The plant extract was documented to exert anti-cancer activity both in vivo and in vitro [2]. Numerous natural steroidal saponins isolated from herbs show potential apoptosis-promoting activity against several cancer cells types [3,4,5]. In addition, *P. polyphylla* treatment can inhibit epithelial–mesenchymal transition (EMT) and invasion in breast cancer [6] and lung cancer cells [3,4,5]. Recently, *P. polyphylla* extract was also found to inhibit ovarian carcinoma cell growth [7].

The use of complementary and alternative medicine is now a very popular option to support conventional therapy in many countries [8,9,10]. For example, many herbal formulas and remedies based on traditional Chinese medicine are well accepted among cancer patients with Chinese background [11,12,13]. Traditional Chinese medicine (TCM) is based on the use of natural products and well-established theoretical approaches. TCM provides many potential candidates as effective drugs for integrated cancer chemotherapy, such as TJ-41 (Bu-Zhong-Yi-Qi-Tang) and PHY906 (Huang-Qin-Tang) [11,12]. In TCM practice, a therapeutic formula is normally prepared as an aqueous extract mixed with various medical herbs. One major herb in this formula is responsible for relieving the target symptom, whereas other medicinal herbs are added to enhance the therapeutic effects or reduce the side effects of the major herb [13].

Colorectal cancer is one of the most common cancer types worldwide with particularly high incidences in developed countries [14]. In Taiwan, colorectal cancer is the most common type of cancer and the third most common cause of cancer-related deaths [15]. Currently, surgery is still the only curative treatment for colorectal cancer. Although 75–80% of newly diagnosed cases are localized or regional tumors, around 50% of patients suffer recurrence after surgery [16,17]. Adjuvant therapy such as postoperative chemotherapy is used to eliminate remaining lesions and help control the risk of recurrence. Chemotherapy is also one of the main treatment approaches in advanced and recurrent cases while often associated with adverse side effects in patients, particularly in the elderly population [12,13]. Various drug resistance problems in colorectal cancer cases also reduce the response rates. These clinical features limit the use of chemotherapy in patients. Any effective drug which promotes the tumor suppression efficacy of chemotherapeutic regimens or eases the associated adverse effects may serve as an appropriate candidate to establish integrated chemotherapy and improve clinical outcomes in cancer patients. Combining standard chemotherapeutics with antitumor drugs to induce tumor cell death via other molecular pathways would not only improve tumor suppression efficiency but also reduce the doses of chemotherapeutic drugs, which could help control adverse effects and may slow the development of drug resistance. Due to the use of chemotherapy as the main approach for advanced and recurrent cancers, developing effective complementary drugs could help improve tumor suppression efficiency and control adverse effects from chemotherapy. DLD-1 is a colorectal adenocarcinoma cell line similar to HT-29 and Caco-2 cell lines [16], which are established from tumorigenic epithelial tissue. In this study, we investigated the effect of the ethanolic extracts of *P. polyphylla* (EEPP) on the suppression of DLD-1 colorectal carcinoma cells with or without chemotherapeutic drug (doxorubicin) treatment.

## 2. Results and Discussion

### 2.1. Treatment Effect of P. polyphylla on Colorectal Cancer Cell Growth

**Figure 1:**
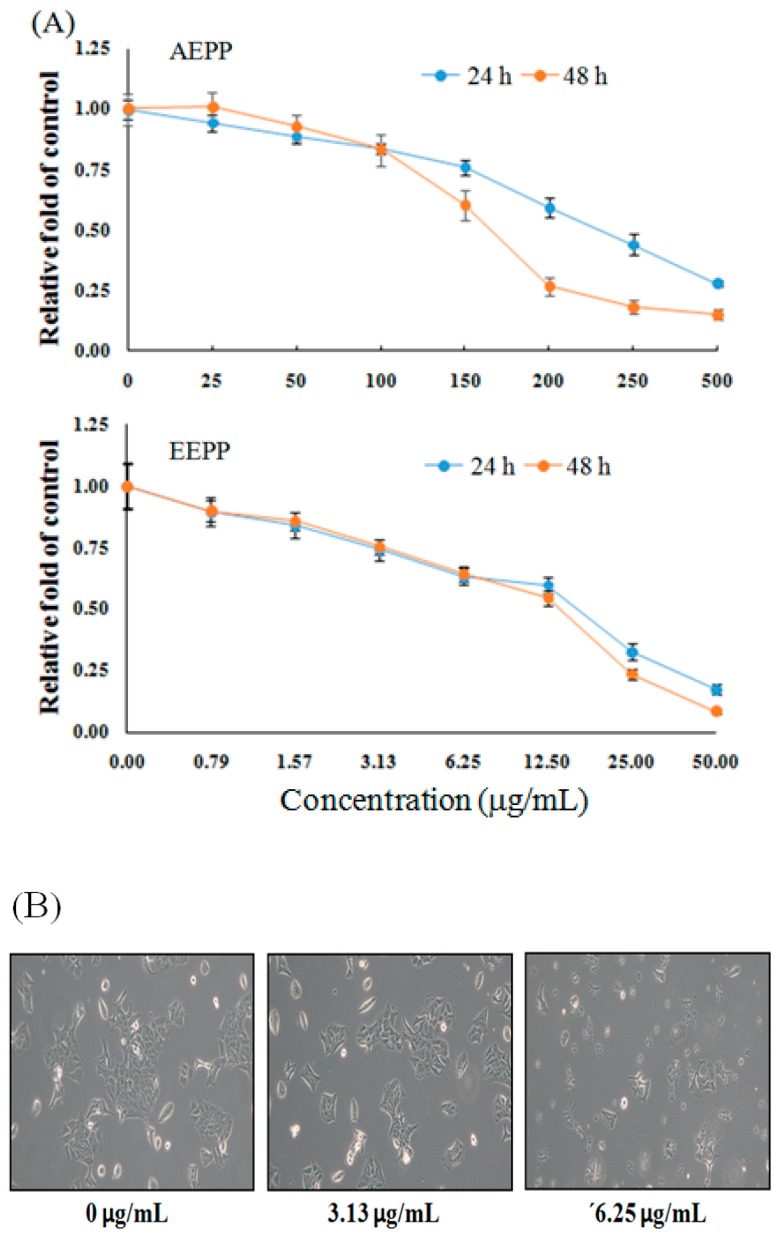
Inhibitory effect of *Paris polyphylla* on colorectal cancer cells. (**A**) Inhibitory effect of aqueous extract of *P. polyphylla* (AEPP) or ethanolic extract of *P. polyphylla* (EEPP) on DLD-1 colorectal carcinoma cells after treatment for 24 and 48 h, respectively. Data are shown as means ± SD (*n* = 3). (**B**) The morphological appearance of DLD-1 colorectal carcinoma cells after 24 h of EEPP treatment.

As shown in Figure 1A, compared to the untreated group, cell viability of DLD-1 colorectal carcinoma cells were decreased after treatment with 3.13–50 μg/mL EEPP for 24 or 48 h in a dose-dependent manner. On the other hand, the aqueous extract of *P. polyphylla* (AEPP) required higher doses to inhibit the growth of colorectal cancer cells. In addition, EEPP treatment, particularly at 6.25 μg/mL, induced apparent morphological alterations in the DLD-1 cells compared to the untreated group (Figure 1B). These results indicate that EEPP treatment induced cytotoxicity in colorectal carcinoma cells, suggesting that EEPP treatment causes DLD-1 colorectal cancer cell death.

**Figure 2:**
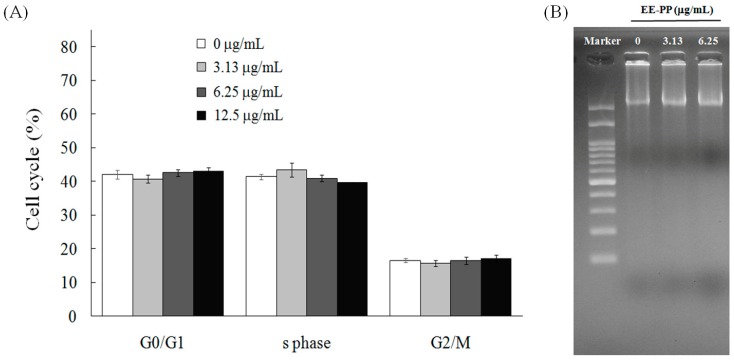
No effects of EEPP on (**A**) cell-cycle distribution and (**B**) DNA ladder in DLD-1 colorectal carcinoma cells. Data are shown as means ± SD (*n* = 3).

One approach in developing integrated chemotherapy is to choose a drug which enhances tumor cell suppression efficiency by increasing cytotoxicity using a different cell death mechanism from the other drugs used in the regimen. In general, the tumor suppression mechanisms of current chemotherapeutic drugs are mainly based on disruption of cell-cycle processes, resulting in cell apoptosis. Next, we sought to examine the possible mechanism through which EEPP causes DLD-1 colorectal cancer cell death. To this end, we examined the effect of EEPP treatment on cell-cycle regulation in the DLD-1 cells. As indicated in Figure 2A, treatment of the DLD-1 cells with 3.13–13.5 μg/mL EEPP for 12 h demonstrated a similar cell-cycle distribution pattern to the control group, suggesting that EEPP does not disrupt the cell-cycle progression in the DLD-1 colorectal cancer cells. To further determine the cell death pathway involved in EEPP-induced colorectal carcinoma cytotoxicity, we tested for DNA fragmentation associated with apoptosis. As indicated in Figure 2B, EEPP treatment did not induce DNA fragmentation in the DLD-1 cells. Together, these results suggest that the EEPP-mediated inhibition of the DLD-1 colorectal cancer cell growth does not involve apoptosis.

### 2.2. EEPP Treatment Causes Autophagic Cell Death in Colorectal Carcinoma Cells

**Figure 3:**
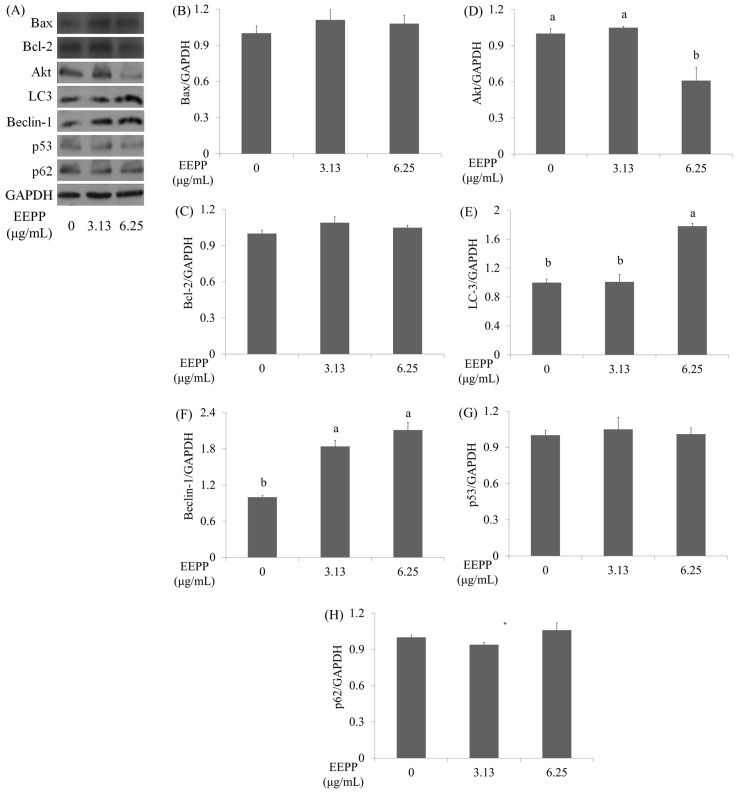
The effects of EEPP on apoptosis and autophagy markers. (**A**) The effects of EEPP on Bax, Bcl-2, Akt, LC-3, Beclin-1, p53, and p62 levels in DLD-1 colorectal carcinoma cells after 24 h of treatment. (**B**–**H**) Quantitative analysis for each protein levels. Data are shown as means ± SD (*n* = 3). The significant differences are denoted by different letters (*p* < 0.05).

Apart from apoptosis, autophagy also plays crucial roles in cancer cell survival and death, and is gaining increasing interest in cancer research. Autophagy, also termed type II programmed cell death (PCD), is a physiologic process that allows sequestration and degradation of the cytoplasmic contents through the lysosomal machinery [18]. Autophagy allows recycling of cellular components and ensures cellular energy supplement during nutrition starvation, infection, and other stress conditions [19]. Several lines of studies suggest cytotoxic agents including chemotherapeutic agents induce cancer cell autophagy [20,21,22]. To investigate whether autophagy is implicated in the EEPP-induced DLD-1 colorectal carcinoma cell death, cells were treated with EEPP for 24 h for evaluating the expression levels of the autophagy-related proteins including Beclin-1, microtubule-associated protein-1 light chain-3 (LC3), and p62 (a marker for autophagic degradation) [23,24], as well as the apoptosis-associated proteins such as Bax (Bcl2-associated X protein), p53 (tumor protein p53), Akt (Protein Kinase B), and Bcl-2 (B-cell lymphoma 2). As shown in Figure 3, in contrast to the untreated control groups, autophagy markers such as LC3 and Beclin-1 proteins were increased after treating with EEPP for 24 h in a dose-dependent manner (Figure 3A, E and F). On the other hand, Akt level was downregulated in EEPP-treated DLD-1 cells after 24 h of treatment (Figure 3A, D), whereas the expression of p62 (Figure 3A, H), and the apoptosis markers such as p53 (Figure 3A, G), Bax (Figure 3A, B), and Bcl-2 (Figure 3A, C) proteins were not affected by EEPP treatment. These results indicated that EEPP treatment induced autophagic cell death in the DLD-1 cells.

### 2.3. Effect of EEPP–Doxorubicin Combination Treatment on Autophagy Induction in Colorectal Carcinoma Cells

**Figure 4:**
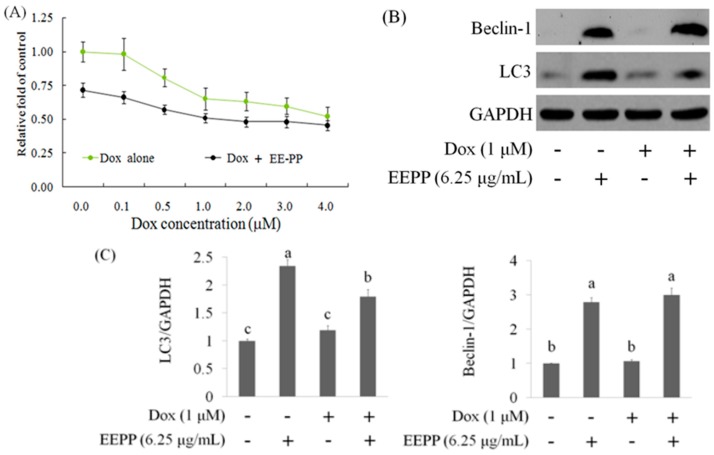
The combined effect of EEPP with doxorubicin (**A**). The suppressive effect of EEPP (3.13 μg/mL) combined with doxorubicin (Dox) for 24 h in DLD-1 colorectal carcinoma cells (**B** and **C**). The upregulation of Beclin-1 and LC3 expressions in EEPP-treated DLD-1 carcinoma cells. Data are shown as means ± SD (*n* = 3). The significant differences are denoted by diferent letters (*p* < 0.05).

Since EEPP induces autophagic cell death in DLD-1 cells, the present study further examined the potential effect of EEPP in combination with the chemotherapeutic drug doxorubicin (Dox) on DLD-1 cells. Dox functions as a topoisomerase II inhibitor and interferes with DNA/RNA synthesis in tumor cells [25]. Colorectal carcinoma cells were treated with various doses of Dox alone or in combination with EEPP for 24 h. Figure 4A illustrates that Dox treatment dose-dependently decreased cell viability in DLD-1 cells. When compared with Dox treatment alone, EEPP (3.13 μg/mL) combined with Dox treatment displayed stronger inhibitory activity against the DLD-1 cells, indicating that EEPP synergizes with Dox to inhibit the DLD-1 colorectal cancer cell growth.

Given that Dox is a well-known chemotherapeutic drug that induces apoptosis via the activation of p53 and caspase-3 signaling pathways in many tumor cells, we speculate that the increased cancer cell death resulting from Dox–EEPP combination treatment could be due to the potentiation of the triggered cell death pathways. To examine this hypothesis, DLD-1 cells were treated with 6.25 μg/mL EEPP alone, 1 μM Dox alone, or Dox in combination with 6.25 μg/mL EEPP for 24 h for Western blot analysis against the apoptosis- and autophagy-related proteins. In contrast to p53 and caspase-3, whose expressions were unaltered by the combination therapy (data not shown), Dox in combination with EEPP increased both LC3 and Beclin-1 protein expressions compared to Dox alone (Figure 4B, C). This concomitant increase in autophagy markers is likely due to the presence of EEPP, which alone also upregulated the autophagy markers. Together, these results suggested that EEPP may potentially enhance the anti-tumor effect in human colorectal carcinoma cells when combined with Dox.

### 2.4. Isolation and Identification of Active Compounds from EEPP

**Figure 5:**
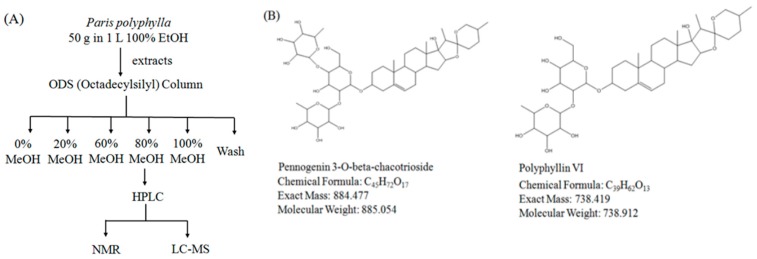
Isolation of active ingredients from EEPP. (**A**) The flowchart for identification of active compounds obtained from EEPP. (**B**) Pennogenin 3-*O*-beta-chacotrioside and polyphyllin VI were isolated and confirmed by NMR and LC–MS.

**Figure 6:**
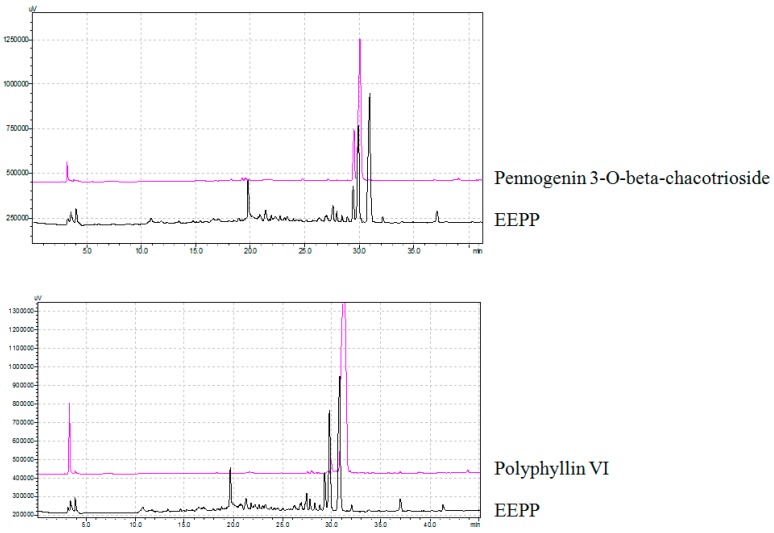
The purity of pennogenin 3-*O*-beta-chacotrioside and polyphyllin VI isolated from EEPP.

After showing that EEPP induces autophagic cell death in DLD-1 cells, we next sought to identify the active components of the extract responsible for its cytotoxicity. We used an octadecylsilyl column to separate EEPP into five fractions by different percentages of methanol elution (Figure 5A), after which the cytotoxicity of the fractions against the DLD-1 cells was tested. Cell viability of DLD-1 colorectal carcinoma cells was decreased after treating with the 80% methanolic fraction for 24 h (0% group: survival at 78.6%; 20% group: survival at 73.7%; 60% group: survival at 58.1%; 80% group: survival at 21.7%; 100% group: survival at 38.7%). We further isolated the active components from the 80% methanolic fraction by LC–MS and confirmed the active compounds by NMR. Pennogenin 3-*O*-beta-chacotrioside and polyphyllin VI were the two main compounds isolated from the 80% methanolic fraction (Figure 5B), with purity up to 95% (Figure 6).

**Figure 7:**
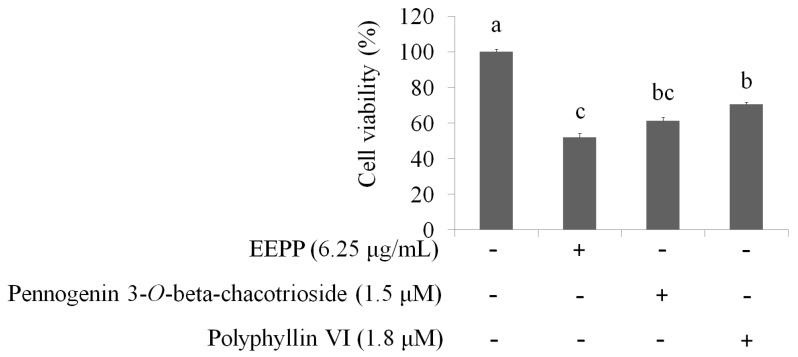
The suppression of DLD-1 colorectal carcinoma cells treated with EEPP (6.25 μg/mL), pennogenin 3-*O*-beta-chacotrioside (1.8 μM), or polyphyllin VI (1.4 μM) for 24 h. The concentrations of pennogenin 3-*O*-beta-chacotrioside and polyphyllin VI were calculated according to EEPP. Data are shown as means ± SD (*n* = 3). The significant differences are denoted by different letters (*p* < 0.05).

Next, the concentrations of pennogenin 3-*O*-beta-chacotrioside and polyphyllin VI were calculated according to EEPP, and the cell viability of DLD-1 colorectal carcinoma cells was determined. The data showed that, when compared to the untreated group, treatment of cells for 24 h with EEPP (6.25 μg/mL), pennogenin 3-*O*-beta-chacotrioside (1.8 μM), or polyphyllin VI (1.4 μM) decreased DLD-1 cell viability, indicating that pennogenin 3-*O*-beta-chacotrioside and polyphyllin VI are the two main active compounds from EEPP involved in colorectal cancer cell inhibition (Figure 7).

**Figure 8:**
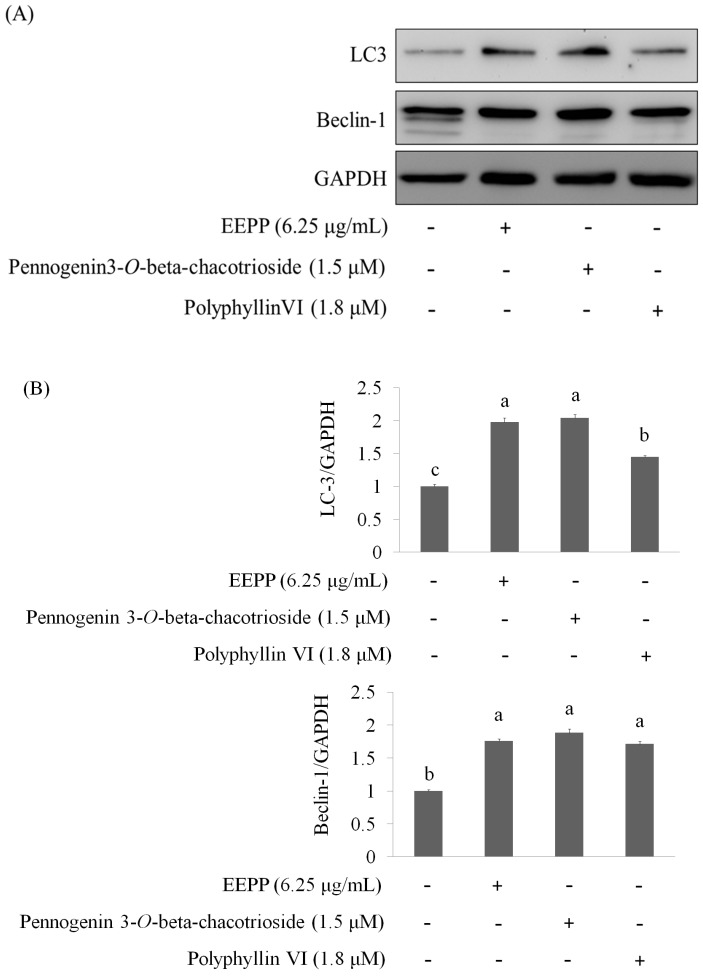
Elevation of autophagy markers in DLD-1 colorectal carcinoma cells treated with pennogenin 3-*O*-β-chacotrioside or polyphyllin VI for 24 h. The concentrations of pennogenin 3-*O*-β-chacotrioside and polyphyllin VI were calculated according to EEPP. (**A**) Pennogenin 3-O-β-chacotrioside or polyphyllin VI markedly increased the expressions of LC3 and Beclin-1. (**B**) Quantitative analysis for each protein levels. Data are shown as means ± SD (*n* = 3). The significant differences are denoted by different letters (*p* < 0.05).

**Figure 9:**
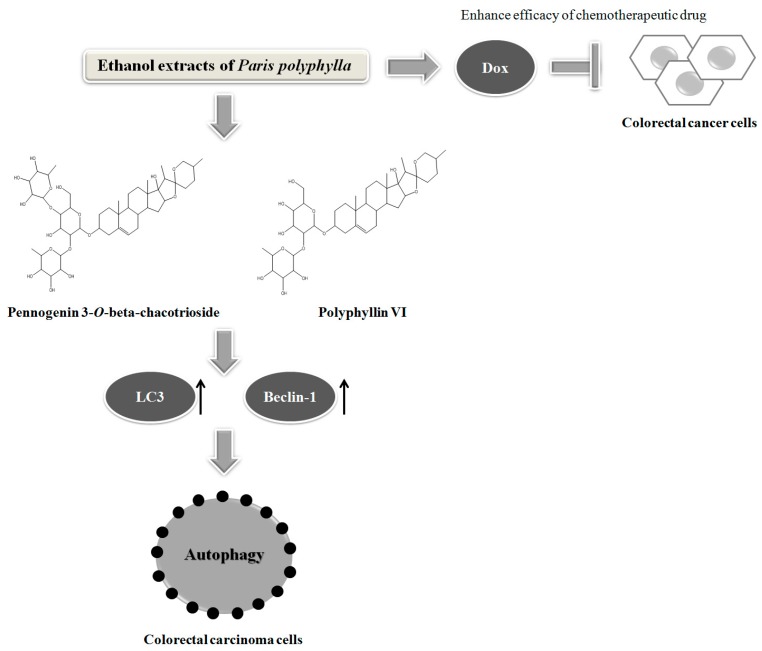
Schematics of the mode of action of *Paris polyphylla* ethanol extract on DLD-1 colorectal cancer cells.

Finally, we asked whether the active components (pennogenin 3-*O*-beta-chacotrioside and polyphyllin VI) could also modulate the expression of the autophagy-related proteins in the DLD-1 cells. As shown in Figure 8A,B, both pennogenin 3-*O*-beta-chacotrioside and polyphyllin VI treatments for 24 h markedly increased the expressions of LC3 and Beclin-1, suggesting that these compounds, similar to EEPP, also inhibit colorectal cancer cell death by modulating autophagy. In conclusion, our results suggest that EEPP deserves further evaluation for development as complementary chemotherapy for colorectal cancer, and pennogenin 3-*O*-β-chacotrioside and polyphyllin VI identified as the main candidate active components in EEPP. Schematics of the mode of action of *Paris polyphylla* ethanol extract on DLD-1 colorectal cancer cells is shown in Figure 9.

## 3. Materials and Methods

### 3.1. Chemicals

*P. polyphylla* was purchased from Taiwan Indigena Botanica Co., Ltd (Taipei, Taiwan), and 10 g of the herb was extracted with ethanol (100 mL) three times at room temperature for 24 h. After evaporating the solvents through freeze-drying, a residue was obtained and stored at −20 °C. Crystal violet, doxorubicin, Propidium iodide (PI), sodium dodecyl sulfate (SDS), Triton X-100, trypsin, and trypan blue were purchased from Sigma Chemical Co. (St. Louis, MO, USA). Fetal bovine serum (FBS) was purchased from Life Technologies (Auckland, New Zealand). Dimethyl sulfoxide was purchased from Wako Pure Chemical Industries (Saitama, Japan). Anti-caspase-3, anti-Bax, anti-Bcl2, anti-p62, anti-p53, anti-LC-3, and anti-GAPDH (Glyceraldehyde 3-phosphate dehydrogenase) antibodies were purchased from Santa Cruz (Santa Cruz, CA, USA). Pennogenin 3-*O*-beta-chacotrioside was purchased from BioCrickBioTech (Chengdu, Sichuan, China). Polyphyllin VI was purchased from Chem Faces (Wuhan, Hubei, China).

### 3.2. Cell Culture

The human colorectal carcinoma cell line DLD-1 (Bioresource Collection and Research Center, HsinChu, Taiwan) was grown in Dulbecco’s modified Eagle’s medium (Gibco BRL, Grand Island, NY, USA) containing 2 mM l-glutamine and 1.5 g/L sodium bicarbonate, supplemented with 10% FBS and 2% penicillin–streptomycin (10,000 U/mL penicillin and 10 mg/mL streptomycin). The cells were cultured in a humidified incubator at 37 °C under 5% CO_2_.

### 3.3. Cell Viability

The cytotoxic effect of EEPP against DLD-1 cells was measured using a crystal violet staining assay. Cells were seeded on 24-well plates (3 × 10^4^ cells per well) and treated with various EEPP concentrations for 24 h. The medium was then removed, washed with phosphate-buffered saline (PBS), stained with 2 g/L crystal violet in phosphate-buffered formaldehyde for 20 min, and washed with water. The crystal violet bound to the cells was dissolved in 20 g/L SDS solution and its absorbance was measured at 600 nm.

### 3.4. Cell Cycle

After 12 h of exposure to 3.13–12.5 μg/mL EEPP, the medium was aspirated and adherent cells were harvested and centrifuged at 300× *g* for 5 min. Cells were washed with PBS, fixed with 700 mL/L ice-cold ethanol at −20 °C overnight, and then stained with PI at room temperature for 30 min. The cell-cycle distribution was analyzed by flow cytometry using an FACScan-LSR flow cytometer equipped with CellQuest software (BD Biosciences, San Jose, CA, USA) [26].

### 3.5. DNA Ladder 

DLD-1 cells were treated with EEPP for 24 h; the cells were then harvested by scraping with a disposable cell lifter, suspended in PBS, and centrifuged for 10 min (250× *g*) at 4 °C, and the pellet was suspended in 0.1 mL of hypotonic lysing buffer (10 mM Tris, pH 7.4; 10 mM EDTA, pH 8.0; 0.5% Triton X-100). The cells were incubated for 10 min at 4 °C, and the resultant lysate was centrifuged for 30 min (13,000× *g*) at 4 °C. The supernatant, which contained fragmented DNA, was digested and incubated for 1 h at 37 °C with 5 mg/mL RNase A and then incubated for 1.5 h at 50 °C with 2.5 mg/mL proteinase K. DNA was precipitated with 0.5 volume equivalent of 10 M ammonium acetate and 2.5-fold volume equivalent of ethanol at −20 °C overnight. The precipitate was centrifuged at 13,000× *g* for 30 min at 4 °C. The resultant pellet was air-dried and resuspended in 10 mM Tris buffer (pH 7.4) containing 1 mM EDTA. An aliquot equivalent to 1 × 10^6^ cells was electrophoresed at 50 V for 1 h in 1.5% agarose gel in 90 mM Tris-borate buffer containing 2 mM EDTA (pH 8.0). After electrophoresis, the gel was stained with ethidium bromide (0.5 μg/mL), and the nucleic acids were visualized with an ultraviolet transilluminator [27].

### 3.6. Western Blot

Cells were rinsed with ice-cold PBS and lysed by RIPA lysis buffer with protease and phosphatase inhibitors for 20 min on ice. Then, the cells were centrifuged at 12,000× *g* for 10 min at 4 °C. Protein extracts (20 μg) were resolved using SDS polyacrylamide gel electrophoresis (SDS-PAGE; 200 V, 45 min). The protein bands were electrotransferred to nitrocellulose membranes (80 V, 120 min). Membranes were then treated with a 5% enhanced chemiluminescence (ECL) blocking agent (GE Healthcare Bio-Sciences) in saline buffer (TBS-T) containing 0.1% Tween-20, 10 mM Tris-HCl, 150 mM NaCl, 1 mM CaCl_2_, and 1 mM MgCl_2_ at a pH of 7.4 for 1 h, and then incubated with the primary antibody overnight at 4 °C. Subsequently, membranes were washed three times in TBS-T and bound antibodies were detected using appropriate horseradish peroxidase-conjugated secondary antibodies, followed by analysis in an ECL plus Western blotting detection system (GE Healthcare Bio-Science) [28].

### 3.7. Method of Isolation and Identification of Active Compounds

Firstly, 50 g of *Paris polyphylla* was dissolved in 1 L of 100% ethanol and extracted. The extracts were then separated using an ODS (octadecylsilyl) column into different parts. After eluting with different concentrations of methanol, 80% methanol-treated parts were isolated and detected by HPLC. Pennogenin 3-*O*-beta-chacotrioside and polyphyllin VI were the two major active compounds in the extracts, identified by LC–MS and NMR.

### 3.8. Statistical Analysis

Results were expressed as means ± SD. Comparisons among groups were made using one-way ANOVA. The differences between mean values in all groups were tested through Duncan’s multiple-range test (SPSS statistical software package, version 17.0, SPSS, Chicago, IL, USA). A *p*-value less than 0.05 was considered as a significant difference between means.

## 4. Conclusions

The present study demonstrated that EEPP induced autophagic cell death in colorectal cancer cells and that EEPP combined with Dox might exert a more potent anti-cancer effect against these tumor cells. We suggest that EEPP and its active ingredients pennogenin 3-*O*-beta-chacotrioside and polyphyllin VI could be further explored as potential candidates for the development of complementary chemotherapy against colorectal cancer.

## Abbreviations

EEPP	ethanolic extract of *Paris polyphylla*
LC-3	light chain-3
TCM	traditional Chinese medicine
PCD	programmed cell death
Dox	doxorubicin
